# Avian Influenza Risk Communication, Thailand

**DOI:** 10.3201/eid1207.060277

**Published:** 2006-07

**Authors:** Masami T. Takeuchi

**Affiliations:** * Food and Agriculture Organization of the United Nations, Rome, Italy

**Keywords:** Avian Influenza, Poultry, Consumer Information, Health Education, Risk Assessment, Thailand, Survey, Food, Safety, letter

**To the Editor:** Twenty-two human cases of H5N1 highly pathogenic avian influenza (HPAI) have been reported in Thailand since 2003, with 14 deaths (*1*). From July to December 2005, I investigated Thai consumers' food safety practices by conducting an oral survey prepared in the Thai language. Interviews were conducted in 3 areas that have not had cases of H5N1 avian influenza, Bangkok (urban, n = 126), Rangsit (suburban, n = 125), and Phetchabun (rural, n = 50). Of the 301 Thai consumers surveyed, 92% thought that Thailand has >1 food safety problems, such as pesticide residues (62%), poor personal hygiene of food vendors (39%), and microbiologic/viral contamination of food (26%). Although the Thai Ministry of Public Health has conducted an aggressive public education campaign regarding HPAI (*2*), only 6% named bird flu as their primary concern. Most participants had some knowledge of avian influenza; 88% of participants knew the name of the disease, and of those, all knew that infections can be deadly, and 97% knew that interacting with and slaughtering infected birds are the most risky activities.

In the rural area, 72% of participants had backyard chickens (almost no one had them in urban and suburban areas). Of those, only 6% were aware of the symptoms of HPAI in poultry. Most villagers knew that minimizing contact with birds could reduce their risk for infection; however, they were not sure how they could minimize contact. None of the owners of backyard chickens had tested them for HPAI. The reporting system for HPAI was not easily accessible for home poultry producers.

The findings of this study are similar to those of Olsen et al., who reported that widespread knowledge of avian influenza had not resulted in behavior change (*2*). Behavior change is a complex process; both motivators and barriers contribute to change. One participant said that the household chickens were a very important economic source, not only for the household but also for her entire village. Eggs were usually consumed within the household or sold at the local market. This villager also said that government educators told villagers not to directly interact with or slaughter chickens at home. Although she was well aware of the danger of HPAI, she thought the recommendations would be impossible to follow since feeding and egg collection involve direct interaction with chickens. When a chicken is no longer able to produce eggs, the participant slaughters the hen and either eats or sells the meat. No facility that could safely slaughter chickens is available in the village, so she does it at home.

The pattern of the villagers' risk perception was interesting. They were very aware of the risk backyard chickens present in the mid-northern area of Thailand, where many HPAI infected poultry have been reported, but they simply thought it would not happen to their chickens. The villagers' lack of concern is compatible with Slovik's theory of risk perception, whereby familiar, naturally occurring risks elicit much less concern than unfamiliar, human-made risks (*3*). The complacency among these villagers indicates that behavior changes will not occur unless villagers are provided with practical recommendations.

Many organizations, such as the Food and Agricultural Organization of the United Nations, the World Health Organization, and the Centers for Disease Control and Prevention, have determined that risk communication is one of the most important strategies to respond to an influenza pandemic. The Thai Ministry of Public Health is conducting a national public awareness campaign to stop the spread of HPAI. Thailand has a rapidly developing metropolitan area and many traditional village areas, and the campaign targets people in all areas. The campaign must provide highly practical recommendations for persons who own backyard chickens.

Three practical items should be included in the campaign: 1) a list of detailed symptoms of HPAI in poultry and humans; 2) guidelines on raising and slaughtering home-raised poultry, with a list of protective equipment such as boots, masks, and goggles, as well as cleaning materials; 3) instructions on how to report sick birds or persons to the Thai Ministry of Health.

Many obstacles prevent Thai consumers from following recommendations to reduce their risk for HPAI, primarily their economic status. Reporting sick birds voluntarily could lead to the destruction of their source of income unless they are compensated for depopulated flocks. To encourage persons to report or test sick birds, home poultry producers should be informed that the Thai government has initiated a system to compensate them for culled birds. Purchasing protective equipment for home slaughter may be cost-prohibitive, however. Therefore, a successful campaign must address economic considerations.

Conducting a risk communication program with consumers can be a tremendous challenge. However, considering the high literacy level of Thai consumers (98%) (*4*), written information is well accepted; therefore, increasing the awareness of HPAI and providing practical recommendations could be achieved in Thailand, if planned carefully.
